# Supplementary material for: External validation of geriatric influenza death score: A multicenter study
Source: PLoS One. 2023 Mar 24;18(3):e0283475. doi: 10.1371/journal.pone.0283475 (PMC10038296; doi:10.1371/journal.pone.0283475)
Supplement: S1 Fig — AUC, area under the curve; GID, Geriatric Influenza Death. (DOCX) [file pone.0283475.s001.docx]

**S1 Figure.** The AUC of GID score. AUC, area under the curve; GID, Geriatric Influenza Death
