# Supplementary material for: External validation of geriatric influenza death score: A multicenter study
Source: PLoS One. 2023 Mar 24;18(3):e0283475. doi: 10.1371/journal.pone.0283475 (PMC10038296; doi:10.1371/journal.pone.0283475)
Supplement: S1 Table — (DOCX) [file pone.0283475.s002.docx]

**S1 Table.** Missing data and their given values

| Variable | Number of missing values | % of total | Given value |
| --- | --- | --- | --- |
| Glasgow coma scale | 318 | 5.8 % | 15 |
| Bandemia (>10% band) | 598 | 10.9 % | 0 % |
| hs-CRP | 2103 | 38.2 % | 2.5 mg/dL |

C-reactive protein, CRP.
